# Nasopharyngeal carriage of Streptococcus pneumoniae in children aged two to five years old in the conjugate vaccine era: A cross-sectional study in Salvador, Brazil

**DOI:** 10.1371/journal.pone.0357903

**Published:** 2026-09-09

**Authors:** Isabela Oliveira Sousa, João Lucas Pinheiro Leite, Amanda Oliveira dos Santos Melo, Adriano Souza Santos Monteiro, Carolina Ferreira Cavalcanti Xavier, Valmira de Jesus Santos, Ana Paula Silva de Lemos, Samanta Cristine Grassi Almeida, Ana Paula de Oliveira Menezes, Soraia Machado Cordeiro, Joice Neves Reis

**Affiliations:** 1 School of Pharmacy, Federal University of Bahia, Ondina, Salvador, Bahia, Brazil; 2 Center of Bacteriology, National Laboratory for Meningitis and Invasive Pneumococcal Infections, Institute Adolfo Lutz, São Paulo, Brazil; 3 State University of Bahia (UNEB), Cabula, Salvador, Bahia, Brazil; George Washington University School of Medicine and Health Sciences, UNITED STATES OF AMERICA

## Abstract

**Background:**

Monitoring the prevalence and serotype distribution of *Streptococcus pneumoniae* nasopharyngeal carriage is essential for understanding transmission dynamics and assessing the impact of pneumococcal conjugate vaccines (PCVs). In Brazil, PCV10-GSK is routinely administered at 2 and 4 months of age with a booster at 12 months. This study aimed to determine the nasopharyngeal carriage rate, serotype distribution, antimicrobial susceptibility, and factors associated with carriage among healthy children aged 2–5 years vaccinated with PCV10-GSK under the Brazilian routine immunization program.

**Methods:**

A cross-sectional study was conducted from August to November 2023 among children aged two to five years from 10 randomly selected schools in Salvador, Brazil. Within each school, all eligible children whose parents or legal guardians provided written informed consent were enrolled. Nasopharyngeal swabs were collected, and demographic, vaccination, and risk factor data were recorded. *S. pneumoniae* isolates were serotyped using multiplex polymerase chain reaction and/or the Quellung reaction. Antimicrobial susceptibility was evaluated using disk diffusion and gradient strip minimum inhibitory concentration methods. Risk factors for carriage were assessed using univariate and multivariable logistic regression analysis.

**Results:**

Among the 400 children enrolled, the overall *S. pneumoniae* carriage rate was 39.5%. White race was independently associated with lower odds of carriage compared with mixed race, whereas none of the evaluated factors was significantly associated with carriage of non-PCV20 serotypes among colonized children. The most frequent serotypes were 6C (17.9%), 19A (13.0%), 11A (9.3%), 15B (8.6%), 23A (8.6%), and 15A (7.4%). Estimated vaccine serotype carriage was 3.2% for PCV10-GSK, 17.3% for PCV13/PCV15/PCV10-SII, and 39.5% for PCV20. Penicillin non-susceptibility was observed in 21.8% of isolates, with the highest rates among serotypes 19A (71.4%), 23A (35.7%), and 6C (20.7%).

**Conclusions:**

A high pneumococcal carriage rate, predominantly involving non-PCV10-GSK serotypes, was observed among children vaccinated under the Brazilian PCV10-GSK program. The limited serotype coverage of PCV10-GSK, together with antimicrobial resistance among circulating serotypes, underscores the need for ongoing surveillance to guide vaccine policy and antimicrobial stewardship in Brazil.

## Introduction

*Streptococcus pneumoniae* remains a leading cause of morbidity and mortality worldwide, particularly among children under five years of age, despite substantial advances in prevention and treatment strategies [1]. The human nasopharynx serves as the primary reservoir for *S. pneumoniae*, playing a critical role in both disease development and transmission within the community [2–4]. Pneumococcal carriage is influenced by several host and environmental factors, including age, daycare attendance, number of siblings, and vaccination status [5,6]. As a result, isolates colonizing the nasopharynx often reflect those circulating strains in the community, some of which may also cause invasive infections.

Over recent decades, the development and global implementation of pneumococcal conjugate vaccines (PCVs) have significantly reduced the burden of invasive pneumococcal disease (IPD) related with the specific serotypes included in the vaccine. These vaccines are highly effective not only in preventing IPD caused by vaccine serotypes but also in reducing nasopharyngeal (NP) carriage of these serotypes [7,8]. Currently, five PCVs are available worldwide for the prevention of IPD in children. These include the 10-valent vaccine (PCV10, Synflorix®, GSK), which targets the serotypes: 1, 4, 5, 6B, 7F, 9V, 14, 18C, 19F, and 23F; the 13-valent vaccine (PCV13, Prevenar13®, Pfizer), which includes all PCV10-GSK serotypes plus serotypes 3, 6A, and 19A; the 10-valent Serum Institute of India vaccine (PCV10-SII PNEUMOSIL®), covering the serotypes:1, 5, 6A, 6B, 7F, 9V, 14, 19A, 19F, and 23F; the 15-valent vaccine (PCV15, Vaxneuvance®, Merck Sharp & Dohme Corp.), which adds 22F and 33F to the PCV13 formulation; and the 20-valent vaccine (PCV20, Prevenar 20, Pfizer Inc.), which expands PVC15 with serotypes 8, 10A, 11A, 12F, and 15B [9,10].

Brazil introduced PCV10-GSK (Synflorix®) into its national childhood immunization program in March 2010. The current schedule recommends a two-dose primary series at 2 and 4 months of age, followed by a booster dose at 12 months. As of 2023, vaccination coverage among children under one year of age reached 83.4% [11]. Several studies have documented the vaccine’s impact on reducing IPD caused by PCV10-GSK serotypes, both in the short and long term. Data from Brazil’s national laboratory-based surveillance system showed an 85.6% reduction in IPD caused by PCV10-GSK serotypes among children aged two months to five years within five years of vaccine introduction [12]. Another study, over a nine-year post-vaccine period, reported an 84.7% decline in overall IPD cases and a 98.0% reduction in IPD due to PCV10-GSK serotypes [13].

Despite this success, data on the impact of PCV10-GSK vaccine on NP carriage remain limited, especially outside Brazil’s Southeast region. Studies from that region have demonstrated a marked decline in vaccine-type carriage, alongside an increase in non-vaccine serotypes, particularly serotypes 6C and 19A, which are frequently associated with resistance to penicillin and ceftriaxone [14–16].

Updated data on pneumococcal carriage in the context of a mature PCV10-GSK immunization program are crucial to inform policy decisions regarding the adoption of newer or higher-valency PCVs. This study aimed to estimate the prevalence of pneumococcal NP carriage among children under five years of age in Salvador, the fifth most populous city in Brazil, located in the country’s Northeast region [17], and to identify factors associated with overall pneumococcal carriage and given the near elimination of PCV10-GSK serotypes, carriage of serotypes not covered by PCV20, which currently predominate in the study population. These findings will contribute to evidence-based policymaking regarding the potential incorporation of expanded-valency pneumococcal conjugate vaccines in Brazil.

## Materials and methods

### Study area and population

The city of Salvador, located in the northeastern Brazil, has an estimated 153,587 children under five years of age and offers 19,838 school placements across 433 schools, including 120 municipal schools distributed across the city’s 10 administrative regions. To ensure geographic representation, all eligible municipal preschools within each administrative region were listed, and one school per region was randomly selected, resulting in a total of 10 participating schools.

A cross-sectional survey was conducted between August 31 and November 9, 2023, among children aged 2–5 years enrolled in the selected schools. During scheduled school visits, all children who met the eligibility criteria were invited to participate. Parents or legal guardians were interviewed on the day of sample collection, and children were enrolled only after written informed consent had been obtained. No additional sampling of children was performed within the selected schools.

### Data and specimen collection

On the day of NP specimen collection, immediately prior to specimen collection, trained study personnel administered a standardized questionnaire to the parent or legal guardian of each participating child to obtain demographic and epidemiologic information, including underlying medical conditions, history of hospitalization, occurrence of upper respiratory tract infection (URTI) during the preceding month, recent antibiotic use (within the previous four weeks), and household environmental exposures, including tobacco smoke. PCV10-GSK vaccination status was verified by reviewing the child’s immunization card, and children were classified according to their compliance with the age-appropriate PCV10-GSK schedule recommended by the Brazilian National Immunization Program.

NP specimens were collected using flocked swabs (Copan, Brescia, Italy). Immediately after collection, swabs were placed into cryotubes containing 1 mL of skim milk–tryptone–glucose–glycerol (STGG) transport medium and transported in a cooler with ice packs. Within four hours of collection, the STGG medium was vigorously vortexed for 20–30 seconds, and the specimens were stored at −70°C until further analysis.

### Laboratory methods

For pneumococcal culture, frozen vials were thawed at room temperature and then vortexed for 20–30 seconds. Aliquots of 200 μl were transferred to 5 mL of TYS broth (Todd-Hewitt broth supplemented with 0.5% yeast extract and 1 mL of rabbit serum) and incubated at 35–37°C for six hours. Subsequently, 10 µl of cultured broth was plated onto sheep blood agar and incubated at 35–37°C in 5% CO_2_. After 18–24 hours, plates were examined for alpha-hemolytic colonies consistent with *Streptococcus* species. Three to four presumptive *S. pneumoniae* colonies were confirmed by optochin susceptibility test (Oxoid, Basingstok, UK) and the bile solubility test. Confirmed isolates were stored at −70°C in 40% sterile glycerin bouillon for further analysis.

Pneumococcal isolates were serotyped using a sequential conventional multiplex polymerase chain reaction (cmPCR) method [18,19], followed by confirmation with the Quellung reaction when required at the Brazilian reference laboratory of the Adolfo Lutz Institute.

Antimicrobial susceptibility was assessed using the disk-diffusion method according to CLSI guidelines [20]. Susceptibility to oxacillin, ceftriaxone, erythromycin, clindamycin, trimethoprim-sulfamethoxazole, vancomycin, and levofloxacin was tested using commercial disks (OXOID, Basingstoke, England). Isolates with an inhibition zone diameter < 20 mm surrounding an oxacillin (1 μg) disk were tested for penicillin and ceftriaxone susceptibility using gradient strips minimum inhibitory concentration (MIC) (E-test, BioMérieux, USA) on 5% sheep-blood Muller Hinton agar. MIC thresholds for non-susceptibility were defined as ≥ 0.12 μg/mL for penicillin, and ≥ 2.0 µg/mL for ceftriaxone. *S. pneumoniae* ATCC 49619 was used as the quality control strain. Multidrug resistance (MDR) was defined as resistance to three or more antimicrobial classes.

### Ethical considerations

The study was approved by the Research Ethics Committee of the School of Farmacy at the Federal University of Bahia (approval number 5.761.664). Prior to enrollment, written informed consent was obtained from parent(s) or guardian(s) for each participating child.

### Data analysis

Data were entered into REDCap (Research Eletronic Data Capture; Vanderbilt University, Nashville, TN) and subsequently exported for analysis. Analyses were performed using R software version 4.4.1.

Categorical variables were summarized as absolute and relative frequencies. Associations between potential risk factors and *S. pneumoniae* nasopharyngeal carriage were assessed using univariate logistic regression. Among colonized children, factors associated with carriage of non-PCV20 serotypes were also evaluated using the same analytical approach. Variables with p < 0.20 in univariate analyses and variables considered epidemiologically relevant a priori were included in the multivariable logistic regression models. Crude and adjusted odds ratios (ORs) with 95% confidence intervals (95% CIs) were estimated. A two-tailed p-value < 0.05 was considered statistically significant. Because children were recruited from 10 schools, a sensitivity analysis using a mixed-effects logistic regression model, including school as a random effect was performed for the multivariable model of overall pneumococcal carriage.

For analyses describing pneumococcal serotype distribution, serotypes were categorized according to their inclusion in existing pneumococcal conjugate vaccines: PCV10-GSK serotypes (1, 4, 5, 6B, 7F, 9V, 14, 18C, 19F, and 23F), PCV13: PCV10-GSK serotypes plus 3, 6A, and 19A, PCV15: PCV13 serotypes plus 22F and 33F, PCV20: PCV15 serotypes plus 8, 10A, 11A, 12F, and 15B, and PCV10-SII: serotypes 1, 5, 6A, 6B, 7F, 9V, 14, 19A, 19F, and 23F. Serotypes related to PCV10-GSK but not directly included in the vaccine (e.g., 6A and 19A) were classified as non-vaccine serotypes.

## Results

Between August and November 2023, all 10 pre-selected schools were visited, and a total of 400 eligible children were enrolled in the study. Participant characteristics are summarized in Table 1. Children aged two to three years represented 16.8% of the total, while those aged three to four and four to five years accounted for 42.8% and 40.5%, respectively. Overall, 50.8% of the participants were male. Regarding race, 16.5% identified as white, 33.3% as black, 50.3% as mixed race. Respiratory tract infections in the preceding week were reported for 58.0% of children, and 22.2% had received antimicrobial treatment in the past month. Vaccination records indicated that 92.0% (368 out of 400) had completed PCV10-GSK Brazilian immunization schedule of two doses plus a booster, while 8.0% had incomplete vaccination. Additionally, 1.5% had received PCV13.

**Table 1:** Demographic and clinical characteristics of the study population according to *Streptococcus pneumoniae* nasopharyngeal carriage status (n = 400).

Characteristics	Number of children n (%)	*S. pneumoniae* carriage		Odds Ratio (95% CI)	P value	Adjusted OR (95% CI)	P value
		**Yes (n = 158)** **n (%)**	**No (n = 242)** **n (%)**				
**Age (years)**							
2-3	67 (16.8)	23 (14.6)	44 (18.2)	Ref		Ref	
3-4	171 (42.8)	69 (43.7)	102 (42.1)	1.29 (0.72–2.36)	0.39	1.30 (0.71–2.43)	0.40
4-5	162 (40.5)	66 (41.8)	96 (39.7)	1.32 (0.73–2.41)	0.37	1.23 (0.67–2.30)	0.52
**Sex**							
Male	203 (50.8)	82 (51.9)	121 (50.0)	1.08 (0.72–1.61)	0.71	1.13 (0.74–1.71)	0.58
Female	197 (49.3)	76 (48.1)	121 (50.0)	Ref		Ref	
**Race**							
White	66 (16.5)	19 (12.0)	47 (19.4)	0.51 (0.27–0.92)	**0.03**	0.47 (0.25–0.87)	**0.02**
Black	133 (33.3)	50 (31.7)	83 (34.3)	0.76 (0.48–1.18)	0.23	0.79 (0.50–1.25)	0.31
Mixed	201 (50.3)	89 (56.3)	112 (46.3)	Ref		Ref	
**Hospital admission** ^**&**^							
Yes	9 (2.3)	1 (0.6)	8 (3.3)	0.19 (0.01–1.03)	0.115	0.69 (0.40–1.18)	0.18
No	391 (97.8)	157 (99.4)	234 (96.7)	Ref			
**Chronic disease**							
Asthma	25 (6.3)	8 (5.1)	17 (7.0)	0.71 (0.28–1.63)	0.43		
Bronchitis	1 (0.3)	0	1 (0.4)				
Rhinitis	32 (8.0)	11 (7.0)	21 (8.7)	0.79 (0.36–1.65)	0.54		
Sinusitis	5 (1.3)	2 (1.3)	3 (1.2)	1.02 (0.13–6.23)	0.98		
Allergy	3 (0.8)	1 (0.6)	2 (0.8)	0.76 (0.04–8.04)	0.83		
Sickle cell disease	2 (0.5)	1 (0.6)	1 (0.4)	1.54 (0.06–39.01)	0.76		
Hypertension	1 (0.3)	0	1 (0.4)				
Heart disease	1 (0.3)	0	1 (0.4)				
**Antibiotics use in the last month**							
Yes	8 (2.0)	27 (17.1)	61 (25.2)	0.61 (0.36–1.01)	0.06	0.65 (0.38–1.08)	0.10
No	312 (78.0)	131 (82.9)	181 (74.8)	Ref		Ref	
**Upper respiratory tract infection in the last week**							
Yes	232 (58.0)	97 (61.4)	135 (55.8)	0.73 (0.42–1.25)	0.27		
No	168 (42.0)	61 (38.6)	107 (44.2)	Ref			
Number of household contacts*							
1–4	338 (84.5)	129 (81.6)	209 (86.4)	Ref			
5–9	62 (15.5)	29 (18.4)	33 (13.6)	1.42 (0.82–2.46)	0.20		
**Household size** ^**$**^							
1–2	42 (10.5)	21 (13.3)	21 (8.7)	Ref		Ref	
3 - 4	118 (29.5)	53 (33.5)	65 (26.9)	0.82 (0.40–1.65)	0.57	0.73 (0.34–1.54)	0.41
5–6	205 (51.3)	71 (44.9)	134 (55.4)	0.53 (0.27–1.04)	0.06	0.50 (0.24–1.02)	0.06
> 6	35 (8.8)	13 (8.2)	22 (9.1)	0.59 (0.23–1.46)	0.26	0.72 (0.27–1.91)	0.51
**Living with elderly people**							
Yes	58 (14.5)	15 (9.5)	43 (17.8)	0.49 (0.25–0.89)	**0.02**	0.47 (0.24–0.90)	**0.03**
No	342 (85.5)	143 (90.5)	199 (82.2)	Ref		Ref	
**Other children < 5 years in household**							
Yes	170 (42.5)	60 (38.0)	110 (45.5)	0.73 (0.49–1.10)	0.14	0.67 (0.43–1.03)	0.07
No	230 (57.5)	98 (62.0)	132 (54.5)	Ref		Ref	
**Exposure to smoking**							
Yes	62 (15.5)	27 (17.1)	35 (14.5)	1.22 (0.70–2.10)	0.48		
No	338 (84.5)	131 (82.9)	207 (85.5)	Ref			
**Complete PCV-10 vaccination schedule**							
Yes	368 (92.0)	143 (90.5)	225 (93.0)	0.72 (0.35–1.50)	0.38		
No	32 (8.0)	15 (9.5)	17 (7.0)	Ref			
**Other PCV vaccine**							
PCV-13	6 (1.5)	3 (1.9)	3 (1.2)	1.54 (0.28–8.42)	0.60		

Legend: ^&^ Hospital admission in the last month; * Number of people living in the household; ^**$**^Number of rooms in the household. Statistically significant association (*p* < 0.05) are indicated in bold.

The overall NP carriage rate was 39.5% (158 out of 400), yielding 162 pneumococcal isolates due to co-colonization detected in four children. Recent antibiotic use, number of household contacts, household size, exposure to tobacco smoke, contact with other children under five years of age, and complete PCV10-GSK vaccination was not significantly associated with pneumococcal carriage. White race was independently associated with lower odds of pneumococcal carriage compared with mixed race in the main multivariable model (adjusted OR = 0.47, 95% CI: 0.25–0.87, p = 0.02), and this association remained similar in the sensitivity analysis using a mixed-effects logistic regression model including school as a random effect (S1 Table). Living with elderly individuals was also associated with lower odds of carriage in the main model (adjusted OR = 0.48, 95% CI: 0.24–0.90, p = 0.03), although this association was attenuated when school was included as a random effect.

The number of enrolled children was relatively balanced across the 10 participating schools, ranging from 32 to 56 children per school (S2 Table). Pneumococcal carriage prevalence varied across schools, ranging from 18.2% to 64.3%. In a sensitivity analysis, the inverse association between white race and pneumococcal carriage remained similar, whereas the association with living with elderly individuals was attenuated (S1 Table).

Among the 158 children colonized with *S. pneumoniae*, 64 (40.5%) carried at least one PCV20 serotype, whereas 94 (59.5%) carried only non-PCV20 serotypes and/or non-typeable isolates (Table 2). In both the univariate and multivariable analyses, no demographic, clinical, household, or vaccination-related factor was significantly associated with carriage of non-PCV20 serotypes. In the adjusted model, children aged 4–5 years had higher odds of carrying non-PCV20 serotypes than those aged 2–3 years, although this association did not reach statistical significance (adjusted OR = 2.54, 95% CI: 0.92–7.22; *p* = 0.07). Similarly, living with another child younger than five years of age was positively associated with carriage of non-PCV20 serotypes, but the association was not statistically significant (adjusted OR = 1.82, 95% CI: 0.90–3.80; *p* = 0.10).

**Table 2:** Factors associated with carriage of non-PCV20 serotypes among children colonized with *Streptococcus pneumoniae* (n = 158).

Characteristics	PCV20 vaccine-type carriage 64 (%)	Non- PCV20-targeted carriage 94 (%)	Crude OR (95% CI)	P value	Adjusted OR (95% CI)	P value
**Age (years)**						
2-3	13 (20.3)	10 (10.6)	Ref			
3-4	29 (45.3)	40 (42.6)	1.79 (0.69–4.75)	0.23	1.97 (0.73–5.47)	0.18
4-5	22 (34.4)	44 (46.8)	2.60 (0.99–7.02)	0.05	2.54 (0.92–7.22)	0.07
**Sex**			Ref		Ref	
Male	31 (48.4)	51 (54.3)	1.26 (0.67–2.39)	0.47	1.42 (0.73–2.79)	0.30
Female	33 (51.6)	43 (45.7)	Ref		Ref	
**Race**						
White	6 (9.4)	13 (13.8)	1.34 (0.48–4.12)	0.59	1.49 (0.52–4.71)	0.47
Black	24 (37.5)	26 (27.7)	0.67 (0.33–1.35)	0.26	0.56 (0.26–1.17)	0.13
Mixed	34 (53.1)	55 (58.5)	Ref		Ref	
**Hospital admission** ^**&**^						
Yes	1 (1.6)	0				
No	63 (98.4)	94 (100.0)				
**Chronic disease**						
Asthma	5 (7.8)	3 (3.2)	0.39 (0.08–1.64)	0.21		
Bronchitis	0	0				
Rhinitis	5 (7.8)	6 (6.4)	0.80 (0.23–2.91)	0.73		
Sinusitis	2 (3.1)	0				
Allergy	0	1 (1.1)				
Sickle cell disease	0	1 (1.1)				
Hypertension	0	0				
Heart disease	0	0				
**Antibiotics use in the last month**						
Yes	10 (15.6)	17 (18.1)	1.19 (0.51–2.89)	0.69		
No	54 (84.4)	77 (81.9)	Ref			
**Upper respiratory tract infection in the last week**						
Yes	38 (59.4)	59 (62.8)	1.15 (0.60–2.21)	0.67		
No	26 (40.6)	35 (37.2)	Ref			
Number of household contacts*						
1–4	53 (82.8)	76 (80.9)	Ref			
5–9	11 (17.2)	18 (19.1)	1.14 (0.50–2.68)	0.76		
**Household size** ^**$**^						
1–2	7 (10.9)	14 (14.9)	Ref			
3 - 4	20 (31.3)	33 (35.1)	0.83 (0.27–2.35)	0.72		
5–6	31 (48.4)	40 (42.6)	0.65 (0.22–1.75)	0.40		
> 6	6 (9.4)	7 (7.4)	0.58 (0.14–2.43)	0.46		
**Living with elderly people**						
Yes	6 (9.4)	9 (9.6)	1.02 (0.35–3.20)	0.97		
No	58 (90.6)	85 (90.4)	Ref			
**Other children < 5 years in household**						
Yes	19 (29.7)	41 (43.6)	1.83 (0.94–3.64)	0.08	1.82 (0.90–3.80)	0.10
No	45 (70.3)	53 (56.4)	Ref		Ref	
**Exposure to smoking**						
Yes	9 (14.1)	18 (19.1)	1.45 (0.62–3.60)	0.41		
No	55 (85.9)	76 (80.9)	Ref			
**Complete PCV-10 vaccination schedule**						
Yes	57 (89.1)	86 (91.5)	1.32 (0.44–3.88)	0.61		
No	7 (10.9)	8 (8.5)	Ref			
**Other PCV vaccine**						
PCV-13	1 (1.6)	2 (2.1)	1.37 (0.13–29.84)	0.80		

Legend: ^&^ Hospital admission in the last month; ^*^ Number of people living in the household; ^$^ Number of rooms in the household.

**Fig 1:**
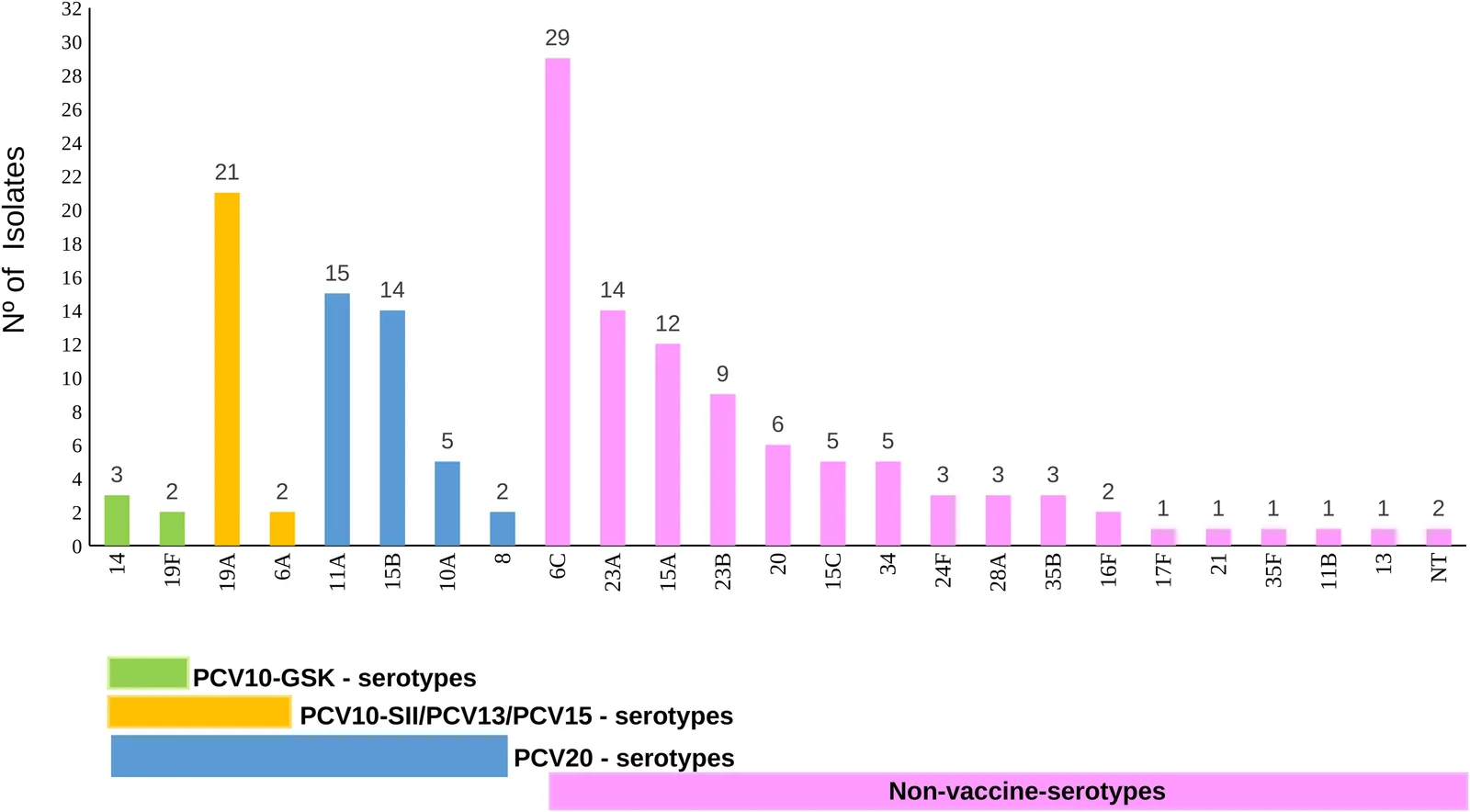
Serotype distribution of *Streptococcus pneumoniae* in the nasopharynx of children aged 2 to 5 years in Salvador, Brazil. PCV10:10-valent vaccine (PCV10, Synflorix®, GSK), which targets the serotypes: 1, 4, 5, 6B, 7F, 9V, 14, 18C, 19F, and 23F; PCV13: the 13-valent vaccine (PCV13, Prevenar13®, Pfizer), which includes all PCV10-GSK serotypes plus serotypes 3, 6A, and 19A; PCV10-SII: the 10-valent Serum Institute of India vaccine (PCV10-SII PNEUMOSIL®), covering the serotypes:1, 5, 6A, 6B, 7F, 9V, 14, 19A, 19F, and 23F; PCV15: the 15-valent vaccine (PCV15, Vaxneuvance®, Merck Sharp & Dohme Corp.), which adds 22F and 33F to the PCV13 formulation; PCV20: the 20-valent vaccine (PCV20, Prevenar 20, Pfizer Inc.), which expands PVC15 with serotypes 8, 10A, 11A, 12F, and 15B.

The most common *S. pneumoniae* serotypes identified among carriers were 6C (17.9%, 29/162), 19A (13.0%, 21/162), 11A (9.3%, 15/162), 15B (8.6%, 14/162), 23A (8.6%, 14/162), and 15A (7.4%, 12/162) (Fig 1). Among the PCV10-GSK vaccine serotypes, 14 (n = 3) and 19F (n = 2) were detected, corresponding to 3.2% of colonized children. Overall, 17.3% (28/162) of circulating serotypes were represented in PCV13, PCV15, or PCV10-SII, whereas 39.5% (64/162) corresponded to serotypes included in PCV20. Co-colonization, presence of more than one pneumococcus serotype, were identified in 2.5% (4/158) of the pneumococcal carriers. The dual-serotype combinations detected were 19A/6C, 19A/20, 14/23A, and 23B/6C.

Antimicrobial susceptibility was available for 160/162 isolates. Oxacillin disk diffusion screening test identified 68 isolates (42.5%) as non-susceptible to penicillin (zone diameter < 20 mm). Among these, seven isolates (4.4%, 7/160) exhibited penicillin resistance (MIC > 2.0 μg/mL), and 28 isolates (17.5%) showed intermediate resistance (MIC 0.125 – < 2.0 μg/mL). Additionally, two isolates (1.3%, 2/160) had ceftriaxone MIC values of 2.0 μg/mL (Table 3).

**Table 3:** Antimicrobial susceptibility of Streptococcus pneumoniae isolated from nasopharyngeal carriage in children aged 2 - 5 years old (n = 160).

Antimicrobial agent	No. (%) of isolates			MIC (µg/mL)		
	Susceptible	Intermediate	Resistant			
Oxacillin	92 (57.5)		68 (42.5)			
Penicillin				**< 0.125**	**0.125-1**	**≥ 2**
				125 (78.1)	28 (17.5)	7 (4.4)
Ceftriaxone				**≤ 1**	**2**	**≥ 4**
				158 (98.7)	2 (1.3)	
						
						
Trimethoprim-sulfamethoxazole	57 (35.6)	13 (8.1)	90 (56.3)			
Levofloxacin	160 (100.0)					
Erythromycin	106 (66.3)		54 (33.8)			
Clindamycin	114 (71.3)		46 (28.8)			
Vancomycin	160 (100.0)					

Footnotes: CLSI, 2024 breakpoints: Penicillin: Susceptible (S) < 0.125 µg/mL, 0.125 µg/mL ≥ Intermediate (I) ≤ 1.0 µg/mL, Resistant (R)>=2 µg/mL; Ceftriaxone: S= ≤ 1 µg/mL, I = 2 µg/mL, R ≥ 4 µg/mL;

Trimethoprim-sulfamethoxazole (SXT) non-susceptibility was common, with 8.1% (13/160) of isolates showing intermediate susceptibility and 56.3% (90/160) exhibiting full resistance. Resistance to erythromycin and clindamycin were observed in 33.8% (54/160) and 28.8% (46/160) of isolates, respectively. All isolates were susceptible to levofloxacin and vancomycin.

Non-susceptibility of individual pneumococcal serotypes to penicillin or ceftriaxone is shown in Table 4. Six serotype 19A isolates and one serotype 11A isolate had a penicillin MIC > 2.0 μg/mL. Among the 28 isolates with a penicillin MIC value between 0.125 and < 2.0 μg/mL, the most frequent serotypes were 19A (n = 9), 6C (n = 6), 23A (n = 5), 14 (n = 3), 15A (n = 2), 10A (n = 2), and 15B (n = 1). Overall, 38 isolates (23.8%) were classified as MDR, with serotypes 19A (n = 15), 6C (n = 8), and 23A (n = 6) accounting for the majority of MDR cases.

**Table 4:** Penicillin and ceftriaxone MIC values according to Streptococcus pneumoniae serotypes isolated from children aged 2–5 years.

Serotype	Number of isolates	Penicillin MIC μg/ml N (%)			Ceftriaxone MIC μg/ml N (%)		
		≤ 0.06	0.125-1	≥ 2	≤ 1	2	≥ 4
**6C**	29	23 (79.3)	6 (20.7)		29 (100.0)		
**19A**	21	6 (28.6)	9 (42.8)	6 (28.6)	20 (95.2)	1 (4.8)	
**23A**	14	8 (57.1)	5 (35.7)		14 (100.0)		
**15A**	12	10 (83.3)	2 (16.7)		12 (100.0)		
**11A**	15	14 (93.3)		1 (6.7)	14 (93.3)	1 (6.7)	
**15B**	14	13 (92.9)	1 (7.1)		14 (100.0)		
**10A**	5	3 (60.0)	2 (40.0)		5 (100.0)		
**14**	3		3 (100.0)		3 (100.0)		
**Total**	113	77	28	7	111	2	

## Discussion

Pneumococcal vaccine coverage in Brazil for the PCV10-GSK was historically high (>90%) within the Unified Health System (SUS) but declined after 2015, because of multiple factors, including declining vaccine confidence, operational challenges in immunization services, and disruptions to routine childhood vaccination during the COVID-19 pandemic, reaching approximately 62% among children under two years of age in 2021. Recovery efforts implemented in 2023–2024, including the National Vaccination Movement, resulted in a partial rebound, with national coverage estimates rising to about 83.1%, although regional disparities persist [11].

In this study involving 400 preschool-aged children enrolled between August and November 2023 in Salvador, Brazil, we observed a pneumococcal carriage rate of 39.5%. Carriage prevalence varied across the participating schools, ranging from 18.2% to 64.3%. This heterogeneity may reflect differences in classroom crowding, socioeconomic conditions, household composition, exposure to respiratory infections, antibiotic use, or local transmission dynamics. In addition, because only one school was sampled per administrative region, the observed variation may also reflect geographic heterogeneity across Salvador.

Notably, only 3.2% of carriage isolates corresponded to serotypes represented in the PCV10-GSK vaccine, underscoring the continued circulation of *S. pneumoniae* among young children in a context of incomplete and recently recovering vaccine coverage and the predominance of non-PCV10-GSK serotypes. Comparable overall pneumococcal nasopharyngeal carriage rates in the post-PCV10-GSK era have been reported in Latin America and the Caribbean, as documented in a recent systematic review and meta-analysis [21]. However, considerable heterogeneity exists across Brazilian regions: studies from Niterói (Southeastern Brazil) reported overall carriage rates of 17.4% [22] and 22.6% [16,21], whereas a recent study from southern Brazil documented an overall carriage prevalence of 63.7% in a similar age group [23]. Together, these findings indicate that nasopharyngeal colonization remains frequent among vaccinated children and continues to represent an important reservoir for pneumococcal transmission and genetic exchange.

Serotype distribution was dominated by non-PCV10-GSK types, particularly serotypes 6C (17.9%), 19A (13.0%), 11A (9.3%), and 15B/15A (16.0% combined). These findings align with previous Brazilian studies, where serotype 6C has emerged as a leading colonizer [15,16,22]. An increasing trend in 6C prevalence has also been reported in countries such as Iceland and Belgium following the replacement of PCV13 with PCV10-GSK, possibly due to the removal of serotypes 6A and consequent loss of cross-protection against 6C that may have been indirectly provided by PCV13 [24,25]. In Brazil, serotype 6C was recently identified as the third most frequent serotype among isolates related with invasive pneumococcal disease in children under five years of age and in the general population [26]. Similar trends have been observed across other South American countries using PCV10-GSK [27]. The emergence of MDR serotype 6C isolates raises additional concerns for both colonization and disease [16].

Environmental and behavioral factors such as household crowding, exposure to tobacco, contact with other young children, and recent antimicrobial use were not significantly associated with overall pneumococcal carriage in the multivariable analysis. Although close contact with young children is a well-established risk factor for pneumococcal carriage, all participants in our study attended preschool, where frequent contact with peers likely represented the predominant source of pneumococcal exposure [28]. Consequently, additional household contact with young children may have contributed little incremental risk. The white race was associated with lower odds of carriage compared with mixed race. Living with elderly individuals was associated with lower odds of carriage in the primary multivariable model. However, this association was attenuated after adjustment for school, suggesting that the initial finding was likely influenced by school-level or geographic confounding rather than representing a true protective effect.

Among children colonized with *S. pneumoniae*, we did not identify demographic, clinical, household, or vaccination-related factors independently associated with carriage of non-PCV20 serotypes. Although older age and living with another child under five years of age showed positive trends in the adjusted analysis, these associations did not reach statistical significance. The absence of clear predictors may reflect the widespread circulation of non-PCV20 serotypes in this highly vaccinated population, suggesting that exposure to these serotypes is no longer restricted to specific demographic or behavioral subgroups. These findings support the hypothesis that non-PCV20 serotypes have become established in the community following the decline of PCV10-GSK vaccine serotypes, reinforcing the importance of continued surveillance to monitor the emergence and dissemination of replacement serotypes.

The high prevalence of serotype 19A, an invasive, antimicrobial resistant type not included in PCV10-GSK vaccine was particularly concerning [29]. This serotype is included in PCV13 and newer, higher-valency vaccines. Consistently, serotype 19A has been detected with increasing frequency in colonization and disease in several Brazilian regions and other countries that have adopted PCV10-GSK [15,24,25,30].

According to national laboratory surveillance data, 19A was the most common serotype associated with IPD in Brazil in 2023, responsible for nearly half of IPD cases in children under five years old and remained the leading cause of IPD across all age groups (26). Similarly, in other Latin American countries that adopted the PCV10-GSK, serotype 19A has also emerged as a leading cause of IPD [27]. These findings emphasize the limited serotype coverage of PCV10-GSK and highlight the potential benefits of introducing broader-valency formulation. In our study, only 17.3% of the identified serotypes were covered by PCV13, PCV15, or PCV10-SII, whereas 39.5% were included in PCV20. At the child level, 40.5% of colonized children carried at least one PCV20 vaccine-type serotype, while 59.5% carried only non-PCV20-targeted serotypes and/or non-typeable isolates.

On the other hand, serotype 3, highly invasive and frequently isolated in IPD was not detected in this carriage study. Although rarely found in healthy children, serotype 3 is a major cause of bacteremia and meningitis in Brazil [29,31,32]. Its low colonization capacity, paired with high disease potential, supports the inclusion of this serotype in future vaccine strategies and the development of formulations capable of eliciting stronger protection against it [33].

The antimicrobial resistance patterns observed in our study was concerning. High levels of resistance to trimethoprim-sulfamethoxazole (56.3%) were consistent with its historical overuse in Brazil and other settings [34]. Resistance to erythromycin (33.8%) and clindamycin (28.8%) was also notable, limiting the effectiveness of empirical therapy. Importantly, 42.5% of isolates were nonsusceptible to penicillin by oxacillin screening, with 4.4% demonstrating high-level penicillin resistance (MIC > 2.0 µg/mL). These findings align with national antimicrobial resistance surveillance data, particularly among serotype 19A and 11A [15,29].

Co-colonization with multiple serotypes was identified in 2.5% of children, including combinations such as 19A/6C and 14/23A. Although infrequent, such events have important epidemiological implications, as they may facilitate horizontal gene transfer and promote the emergence of recombinant isolates [35,36], including those with enhanced virulence or resistance characteristics.

Despite the valuable insights provided, this study has some limitations. First, the cross-sectional design precludes establishing causal relationships between risk factors and pneumococcal carriage. Second, the potential underreporting of antibiotic use by caregivers may have led to misclassification and biased the observed associations. Another limitation is that only one school was sampled within each administrative region. Although this approach ensured geographic representation across Salvador, pneumococcal carriage prevalence varied substantially between schools, suggesting that unmeasured school-level characteristics may have influenced transmission dynamics. A sensitivity analysis including school as a random effect was performed for the multivariable model of overall carriage; however, residual confounding by unmeasured school-level factors cannot be excluded. Therefore, the findings may not fully capture the variability in carriage patterns among all preschools in the city.

Overall, our findings reinforce the importance of ongoing pneumococcal surveillance in pediatric populations, particularly in the context of widespread vaccine use, shifting serotype dynamics, and rising antimicrobial resistance. The observed serotype distribution, predominance of non-PCV10-GSK and non-PCV20-targeted carriage among colonized children, and resistance patterns suggest that higher-valency vaccines could offer improved protection in these settings. The data offer valuable insights into current carriage dynamics and resistance patterns in a highly vaccinated pediatric population.

## Supporting information

S1 Table Sensitivity analysis of factors associated with overall *Streptococcus pneumoniae* nasopharyngeal carriage including school as a random effect. (DOCX)

S2 Table Distribution of enrolled children and pneumococcal carriage by school. (DOCX)

S3 Data Study dataset. De-identified participant-level data used for the analyses.
(XLSX)
