# Supplementary material for: Nasopharyngeal carriage of Streptococcus pneumoniae in children aged two to five years old in the conjugate vaccine era: A cross-sectional study in Salvador, Brazil
Source: PLoS One. 2026 Sep 9;21(9):e0357903. doi: 10.1371/journal.pone.0357903 (PMC13557334; doi:10.1371/journal.pone.0357903)
Supplement: Supplementary file 1 [file pone.0357903.s001.docx]

**S1 Table. Sensitivity analysis of factors associated with overall *Streptococcus pneumoniae* nasopharyngeal carriage including school as a random effect**

| **Characteristics** | **Adjusted OR (95% CI)** | **P value** |
| --- | --- | --- |
|  |  |  |
| **Age (years)** |  |  |
| 2-3 | Ref |  |
| 3-4 | 1.34 (0.71–2.54) | 0.366 |
| 4-5 | 1.29 (0.68–2.45) | 0.438 |
| **Sex** |  |  |
| Male | 1.07 (0.69–1.65) | 0.763 |
| Female | Ref |  |
| **Race** |  |  |
| White | 0.48 (0.25–0.91) | 0.024 |
| Black | 0.74 (0.46–1.21) | 0.230 |
| Mixed | Ref |  |
| **Hospital admission^&^** |  |  |
| Yes | 0.31 (0.03–2.78) | 0.295 |
| No | Ref |  |
| **Antibiotics use in the last month** |  |  |
| Yes | 0.66 (0.38–1.15) | 0.142 |
| No | Ref |  |
| **Household size^$^** |  |  |
| 1 – 2 | Ref |  |
| 3 - 4 | 0.63 (0.28–1.39) | 0.251 |
| 5 – 6 | 0.41 (0.19–0.87) | 0.021 |
| > 6 | 0.68 (0.24–1.88) | 0.454 |
| **Living with elderly people** |  |  |
| Yes | 0.52 (0.26–1.04) | 0.066 |
| No | Ref |  |
| Other children < 5 years in household |  |  |
| Yes | 0.57 (0.36–0.90) | 0.017 |
| No | Ref |  |

Legend: ^&^ Hospital admission in the last month; ^$^ Number of rooms in the household.
