# Supplementary material for: Nasopharyngeal carriage of Streptococcus pneumoniae in children aged two to five years old in the conjugate vaccine era: A cross-sectional study in Salvador, Brazil
Source: PLoS One. 2026 Sep 9;21(9):e0357903. doi: 10.1371/journal.pone.0357903 (PMC13557334; doi:10.1371/journal.pone.0357903)
Supplement: Supplementary file 2 [file pone.0357903.s002.docx]

**S2 Table. Distribution of enrolled children and pneumococcal carriage by school.**

| School number | Enrolled children  n (%) | *S. pneumoniae* carriage  n (%) | No *S. pneumoniae* carriage  n (%) |
| --- | --- | --- | --- |
| School 01 | 40 (10.0) | 14 (35.0) | 26 (65.0) |
| School 02 | 34 (8.5) | 13 (38.2) | 21 (61.8) |
| School 03 | 41 (10.2) | 19 (46.3) | 22 (53.7) |
| School 04 | 44 (11.0) | 8 (18.2) | 36 (81.8) |
| School 05 | 56 (14.0) | 36 (64.3) | 20 (35.7) |
| School 06 | 37 (9.2) | 17 (45.9) | 20 (54.1) |
| School 07 | 40 (10.0) | 15 (37.5) | 25 (62.5) |
| School 08 | 32 (8.0) | 11 (34.4) | 21 (65.6) |
| School 09 | 36 (9.0) | 12 (33.3) | 24 (66.7) |
| School 10 | 40 (10.0) | 13 (32.5) | 27 (67.5) |
